# The Oral Microbiome in the Elderly With Dental Caries and Health

**DOI:** 10.3389/fcimb.2018.00442

**Published:** 2019-01-04

**Authors:** Qian Jiang, Jia Liu, Liang Chen, Ning Gan, Deqin Yang

**Affiliations:** ^1^College of Stomatology, Chongqing Medical University, Chongqing, China; ^2^Chongqing Key Laboratory of Oral Diseases and Biomedical Sciences, Chongqing, China

**Keywords:** microbiota, aged, dental caries, high-throughput nucleotide sequencing, dental plaque, saliva, biodiversity

## Abstract

With the aging of the population, dental caries in the elderly has received increasing attention. A comprehensive study of the oral microbiome is required to understand its polymicrobial etiology. The results of previous studies are limited and remain controversial. In this study, subjects 60 years and older with and without caries were recruited. Unstimulated saliva and dental plaque were collected from each subject and the bacterial 16S rDNA was amplified using PCR and sequenced by Illumina MiSeq high-throughput sequencing. A total of 92 samples were collected from 24 caries patients and 22 healthy controls. Sequences clustered into 147,531 OTUs, representing 16 phyla, 29 classes, 49 orders, 79 families, 149 genera, and 305 species. All predominant phyla, including *Proteobacteria, Bacteroidetes, Firmicutes, Fusobacteria, Actinobacteria*, and *Saccharibacteria*, were largely consistent in different groups, but different relative abundances could be observed. The core microbiome was defined with 246 shared species among groups, which occupied 80.7% of all the species detected. Alpha diversity showed no significant differences in bacterial richness or diversity between caries patients and healthy controls, but distinction existed between samples collected from dental plaque and saliva. Beta diversity analysis was performed by PCoA and hierarchical clustering analysis, showing similar results that microorganisms vary between the two niches. The biomarkers of different groups were defined by LEfSe analysis to identify potential caries-related and health-related bacteria. The co-occurrence analysis of the predominant genera revealed significant interactions among oral microbiota and exhibited more complex and aggregated bacterial correlations in caries-free groups. Finally, the functional prediction of the microbiota present in oral samples was performed by PICRUSt, indicating vigorous microbial metabolism in the oral bacterial community. Our study provides thorough knowledge of the microbiological etiology of elderly individuals with caries and is expected to provide novel methods for its prevention and treatment.

## Introduction

The past several decades have seen a remarkable increase in the proportion of the aging population. It has been estimated that by 2050, the number of people over 65 years of age will reach 1.5 billion. Projected population trends make greater demands on oral health care for the aged. For the elderly, reduction of salivary flow rate, development of systemic diseases, changes in local conditions, insufficient oral care, and many other unfavorable factors may result in different kinds of oral diseases (Lacoste-Ferré et al., 2013). Among them, dental caries, periodontal disease, and oral mucosal diseases are the most common.

Dental caries is a chronic progressive infectious disease in humans and is the most prevalent oral disease that greatly affects people's lives. It is estimated by the World Health Organization that almost all adults will suffer from dental caries at some point in their lives. The Global Burden of Disease Study 2016 showed that caries of permanent teeth had the greatest prevalence and the second highest incidence of disease in the whole world (GBD 2016 Disease and Injury Incidence and Prevalence Collaborators, 2017). With the aging of the population, dental caries in the elderly has received increasing attention. The etiological study and ecological prevention of dental caries in the elderly have gradually become a significant oral health issue.

As is generally acknowledged, when the hard tissue of teeth is dissolved by the acid produced by bacteria, dental caries begins to appear. Human beings are regarded as “superorganisms” inhabited by at least 10 times more microbes than our own cells (Gill et al., 2006). The oral cavity is confirmed to be one of the most diverse microbial habitats in the human body, which harbors hundreds of microorganisms including different species of bacteria, viruses, fungi, mycoplasma, and chlamydia (Chen and Jiang, 2014). The microbial flora of the human oral cavity plays an essential role in maintaining oral homeostasis and preventing humans from dental caries and other oral diseases.

Due to the continued advancement of molecular biology technique, an increasing number of culture-independent methods have emerged to better understand how the oral microbiome is associated with oral health and disease. High-throughput techniques have become efficient approaches for oral microbial analysis (McLean, 2014).

Previous studies have discovered that *Streptococcus mutans, Lactobacilli*, and members of the genera *Bifidobacterium, Actinomyces, Propionibacterium, Veillonella*, and *Scardovia* are caries-associated (Becker et al., 2002; Munson et al., 2004; Downes et al., 2011; Tanner et al., 2011; Kaur et al., 2013). However, earlier studies aimed at caries-related microecology were mostly focused on children, adolescents and adults, and studies about the polymicrobial etiology of dental caries in the elderly are incomplete, and the results remain controversial.

Thus, the current study concentrates on comprehensively investigating the diversity and structure of microbial community in the saliva and dental plaque of the elderly with and without dental caries by Illumina MiSeq sequencing. Our study provides a basis for a better understanding of the microbiological etiology of caries in the elderly and novel methods for prevention and treatment.

## Materials and Methods

### Subjects Selection

Caries patients and healthy controls were recruited from the Affiliated Hospital of Stomatology of Chongqing Medical University in Chongqing, China. The inclusion criteria of subjects were as follows: (i) age over 60, (ii) at least 20 existing natural teeth, (iii) good oral hygiene with no bad eating habits, (iv) no other bacterial infectious oral disease, (v) no removable partial denture, bridge, or implant, (vi) no systemic diseases, and (vii) no antibiotic use within 2 months. All of the participants received a comprehensive oral examination, which provided a professional assessment from a specialized dentist based on the standards of the World Health Organization “Oral health surveys: basic methods−5th ed (World Health Organization, 2013).” In accordance with the DMFT index, subjects were divided into caries-active group (DMFT ≥ 6) and caries-free group (DMFT = 0). Twenty-four caries-active subjects and 22 caries-free subjects were finally selected. These participants were sufficiently informed about the aims of the research and provided written informed consent according to the recommendations of the Ethics Committee of Chongqing Medical University.

### Sample Collection

All of the participants were required to avoid eating, drinking, or brushing their teeth 2 h before taking samples and to rinse their mouth with sterile water. For the caries-active group, plaque samples were collected from each caries site. And plaque samples from the caries-free group were collected from healthy surfaces. All supragingival plaque specimens were scraped by sterile Gracey curettes, pooled into sterile 1.5 ml microcentrifuge tubes and immediately stored at −80°C. In addition, unstimulated saliva was also collected and transferred to sterile 1.5 ml microcentrifuge tubes and then frozen in the laboratory until further processing.

### DNA Extraction and PCR Amplification

Based on the manufacturer's protocol, microbial DNA was extracted from all the specimens by the E.Z.N.A® Soil DNA Kit (Omega Biotek, Norcross, GA, U.S.). A NanoDrop 2,000 UV VIS spectrophotometer (Thermo Scientific, Wilmington, USA) was used to test the concentration and purification of the final DNA, and 1% agarose gel electrophoresis showed the DNA quality. Primers 338F (5′-ACTCCTACGGGAGGCAGCAG-3′) and 806R (5′-GGACTACHVGGGTWTCTAAT-3′) were used to amplify the V3-V4 hypervariable regions of the bacterial 16S rRNA gene using PCR on a thermocycler (GeneAmp 9700, ABI, USA). PCR was conducted using the following program: initial denaturation at 95°C for 3 min, 27 cycles of denaturation at 95°C for 30 s, annealing at 55°C for 30 s, elongation at 72°C for 45 s, and a final extension at 72°C for 10 min. PCR was performed in triplicate with a 20 μL mixture containing 2.5 μL of 10× buffer, 2 μL of 2.5 mM dNTPs, 0.8 μL of each primer (5 μM), 0.2 μL of rTaq Polymerase, 0.2 μL BSA, and 10 ng of template DNA. The final PCR products were extracted from a 2% agarose gel, purified by an AxyPrep DNA Gel Extraction Kit (Axygen Biosciences, Union City, CA, USA) and quantified using QuantiFluor™-ST (Promega, USA) based on the manufacturer's protocol.

### Illumina MiSeq Sequencing

Based on the standard scheme by Majorbio Bio-Pharm Technology Co. Ltd. (Shanghai, China), purified amplicons were merged into equimolar concentrations and paired-end sequenced (2 × 300) on an Illumina MiSeq platform (Illumina, San Diego, USA). The raw reads were deposited into the NCBI Sequence Read Archive (SRA) database (Accession Number: SRP165148).

### Processing of Sequencing Data

Primary FastQ files were divided into multiple files for processing and quality-filtered by Trimmomatic. The following standards were used for sequence combination: (i) The reads were cut off when meeting any site with an average quality score <20 over a 50 bp sliding window. (ii) The primers were perfectly matched allowing the mismatch of two nucleotides, and reads with ambiguous bases were not allowed. (iii) Sequences with overlap longer than 10 bp were merged based on their overlap sequence.

By UPARSE (version 7.1 http://drive5.com/uparse/), operational taxonomic units (OTUs) were clustered with 97% similarity cutoff. Additionally, chimeric sequences were recognized and deleted by UCHIME. By comparing the RDP Classifier algorithm (http://rdp.cme.msu.edu/) against the Silva (SSU123) 16S rRNA database, the taxonomy of each 16S rRNA gene sequence was analyzed based on a 70% confidence threshold.

### Bioinformatics and Statistical Analysis

The bioinformatics analysis was conducted using QIIME (version 1.9.1) (Caporaso et al., 2010). The alpha diversity indices of Shannon, Simpson, Chao, and ACE were calculated at 97% identity by Mothur software (version 1.31.2). Samples from different groups were compared by Student's *t*-test. Beta diversity analysis was performed by principal coordinates analysis (PCoA) based on Bray-Curtis distances at the OTU level. A hierarchical clustering analysis based on weighted UniFrac distances was also conducted (Lozupone et al., 2007). The analysis of similarities (ANOSIM) based on unweighted UniFrac distances was conducted to compare different groups. The Wilcoxon rank-sum test was used to compare the relative abundance of predominant bacteria between different groups. A Venn diagram was used to define the core microbiome at the species level using Mothur (Zaura et al., 2009). Linear discriminant analysis (LDA) of effect size (LEfSe) was conducted to define the biomarkers of the four groups. The threshold on the logarithmic LDA score for distinguishing features was set to 3.0 (Segata et al., 2011). Using Mothur software, co-occurrence analysis among the 20 richest genera was performed. The phylogenetic investigation of communities by reconstruction of unobserved states (PICRUSt, version 1.0.0) program was used to predict 16S rRNA-based data from high-throughput sequencing and to further analyze them in the context of the Cluster of Orthologous Groups (COG) database (Langille et al., 2013). Differences were considered significant when *P* < 0.05 and extremely significant when *P* < 0.01. SPSS 25.0 software (SPSS Inc, Chicago, IL, USA) was used for statistical analysis. The raw data will be made available by the authors, without undue reservation, to any qualified researcher.

## Results

### Sequences Information

All samples were divided into four groups: (1) CP (samples from dental plaque of caries-free subjects, *n* = 22); (2) TP (samples from dental plaque of caries-active subjects, *n* = 24); (3) CS (samples from saliva of caries-free subjects, *n* = 22); and (4) TS (samples from saliva of caries-active subjects, *n* = 24). After Illumina sequencing, a total of 1,740,207 valid sequences were acquired from 92 samples, with 18,915 sequences per sample on average. The mean sequence length was 442 bp, with the longest being 530 bp and the shortest being 235 bp. The clustering of qualified sequences at 97% identity resulted in 147,531 OTUs (Appendix 1). Good's coverage of the generated OTUs reached up to 99.86% and the rarefaction curves (Appendix Figure 1) achieved the even stage, suggesting that the sampling was almost complete.

### Alpha Diversity

The alpha diversity indices of Shannon, Simpson, Chao, and ACE were calculated to analyze the diversity and richness of all the samples. Samples from the caries-active group and caries-free group were compared by Student's *t*-test as well as samples gathered from dental plaque and saliva. There was no significant difference between CP and TP (Table 1A), or between CS and TS (Table 1B), indicating that the diversity and richness of the bacterial communities in caries-active groups were similar to those in caries-free groups. However, by comparing samples of dental plaque and saliva (Table 1C), the indices of Simpson, Chao, and ACE were significantly different (*P* < 0.01), proving that samples collected from dental plaque had higher bacterial diversity and lower richness than saliva.

**Table 1:** Alpha diversity indices of different groups.

**Group**	**Shannon**	**Simpson**	**Chao**	**ACE**
**A**				
CP	3.70 ± 0.30	0.05 ± 0.01	197.64 ± 20.60	200.03 ± 26.66
TP	3.72 ± 0.28	0.05 ± 0.02	189.11 ± 37.97	186.33 ± 35.88
**Group**	Shannon (^*^)	**Simpson**	**Chao**	**ACE**
**B**				
CS	3.47 ± 0.43	0.07 ± 0.03	215.36 ± 35.77	211.61 ± 34.48
TS	3.70 ± 0.31	0.06 ± 0.02	229.77 ± 34.13	225.90 ± 31.22
**Group**	**Shannon**	Simpson (^**^)	Chao (^**^)	ACE (^**^)
**C**				
CP&TP	3.71 ± 0.29	0.05 ± 0.01	193.19 ± 30.88	192.88 ± 32.21
CS&TS	3.59 ± 0.39	0.07 ± 0.03	222.89 ± 35.27	219.08 ± 33.22

Each value is the mean ± SD.

* Represents a significant difference (P < 0.05) and

** *Represents extremely different (P < 001) between groups*.

### Bacterial Community Structure

**Figure 1:**
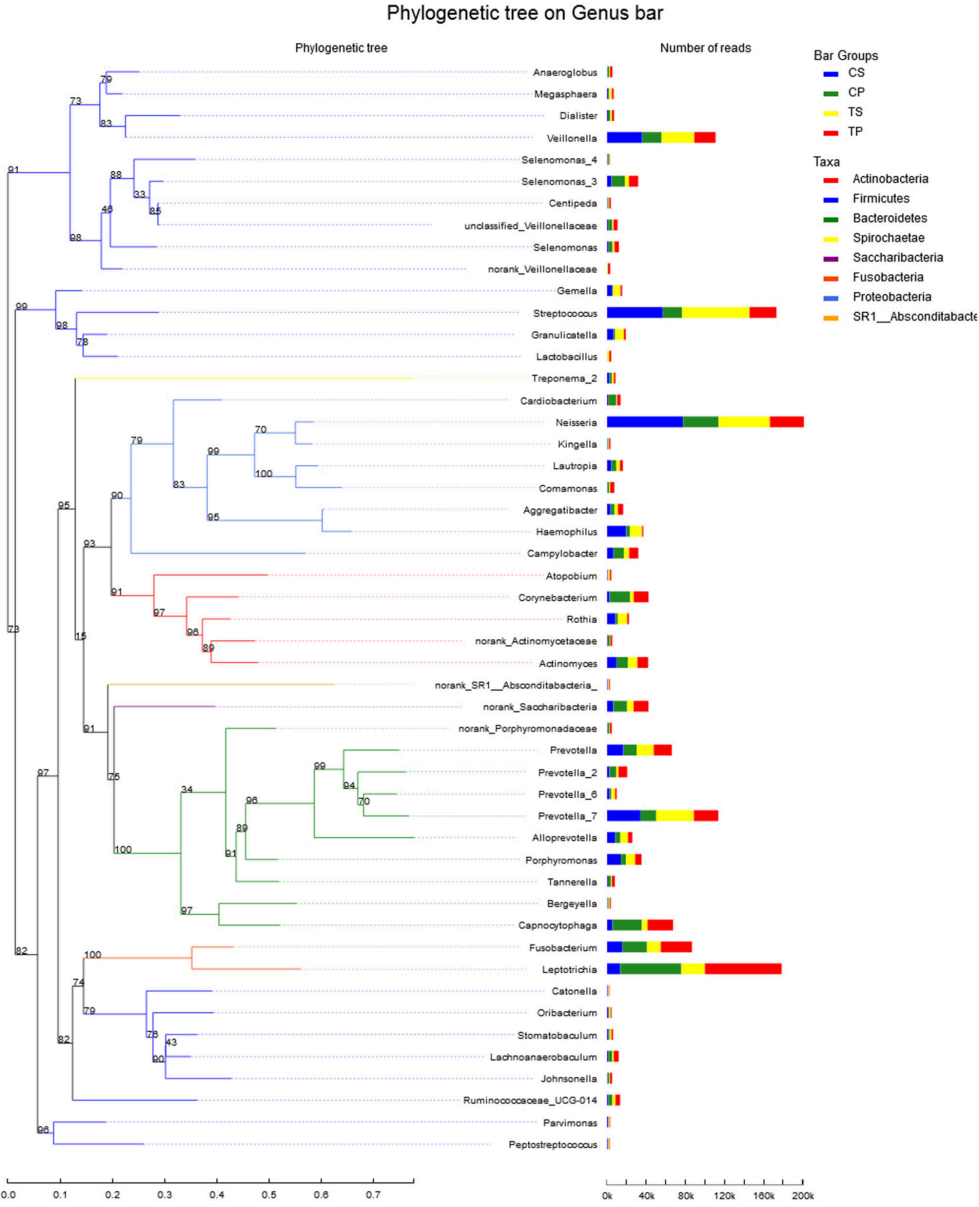
The phylogenetic tree of the 50 most abundant genera. Each branch represents a taxon, the length shows phylogenic distances between two taxa, and different colors represent different phyla. The bar plot on the right side shows the relative abundance of each genus in four groups.

**Figure 2:**
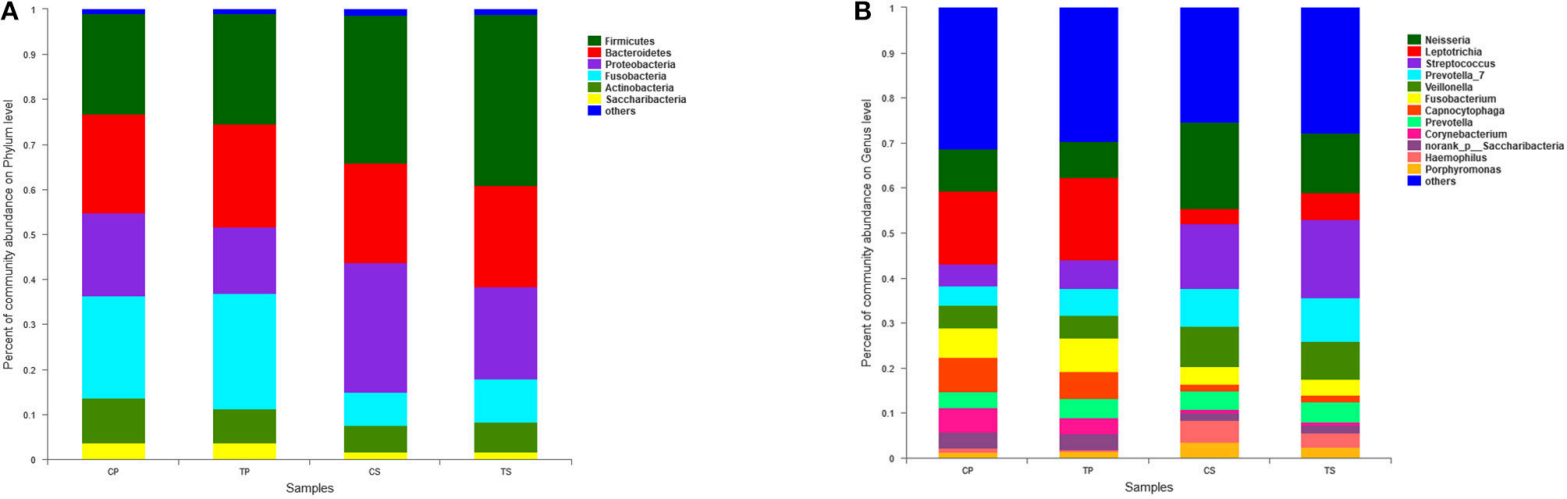
The distributions of the predominant bacteria. **(A)** Results at the phylum level. **(B)** Results at the genus level. The predominant taxa (relative abundance >2% on average) are shown.

**Figure 3:**
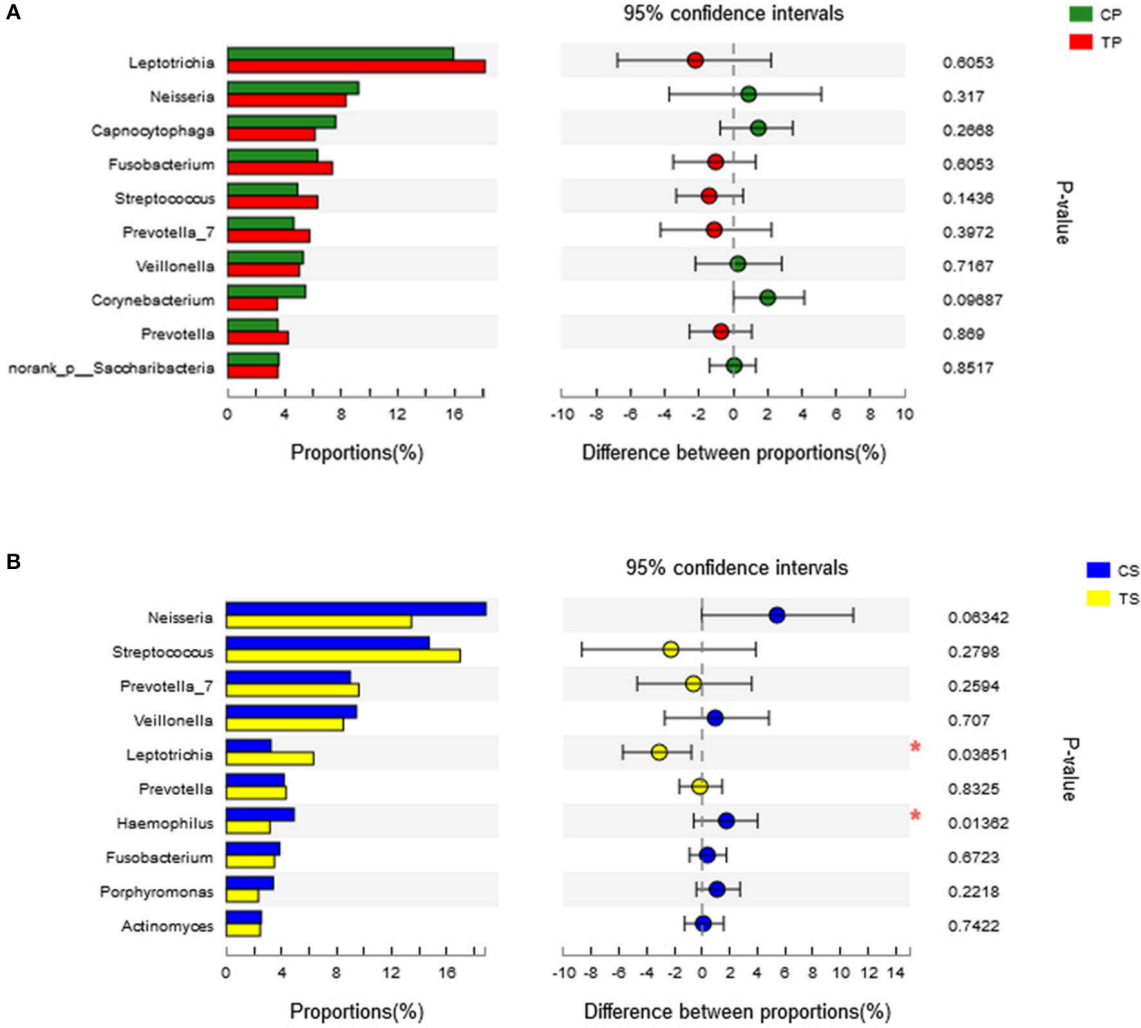
Wilcoxon rank-sum test bar plot on genus level. **(A)** Results of the CP and TP groups. **(B)** Results of the CS and TS groups. ^*^Represents a significant difference (*P* < 0.05).

A total of 16 phyla, 29 classes, 49 orders, 79 families, 149 genera, and 305 species were detected from all the samples. The phylogenetic tree of the 50 most abundant genera was constructed, in which the taxonomy composition and abundance could be observed (Figure 1). The six most abundant phyla were *Firmicutes* (29.6%), *Bacteroidetes* (22.4%), *Proteobacteria* (20.4%), *Fusobacteria* (16.2%), *Actinobacteria* (7.6%), and *Saccharibacteria* (also known as *Candidate division TM7*) (2.6%), together comprising 98.8% of the total sequences. The most prevalent genera were *Neisseria* (12.5%), *Leptotrichia* (11.1%), *Streptococcus* (10.7%), *Prevotella_7* (7.0%), *Veillonella* (6.9%), *Fusobacterium* (5.4%), *Capnocytophaga* (4.2%), *Prevotella* (4.1%), *Corynebacterium* (2.6%), *norank_p_Saccharibacteria* (2.6%), *Actinomyces* (2.6%), *Haemophilus* (2.3%), and *Porphyromonas* (2.2%), totally occupying 74.1% of the whole (Figure 2). The predominant bacteria were largely consistent among the four groups, but different relative abundances could be observed. Comparing relative abundance of the 10 richest genera between caries-active and caries-free subjects, no significant difference was observed between the CP and TP groups. However, discrepancies were detected between the CS and TS groups, with a higher abundance of *Haemophilus* in the CS group and a higher abundance of *Leptotrichia* observed in the TS group (*P* < 0.05, Wilcoxon rank-sum test) (Figure 3).

**Figure 4:**
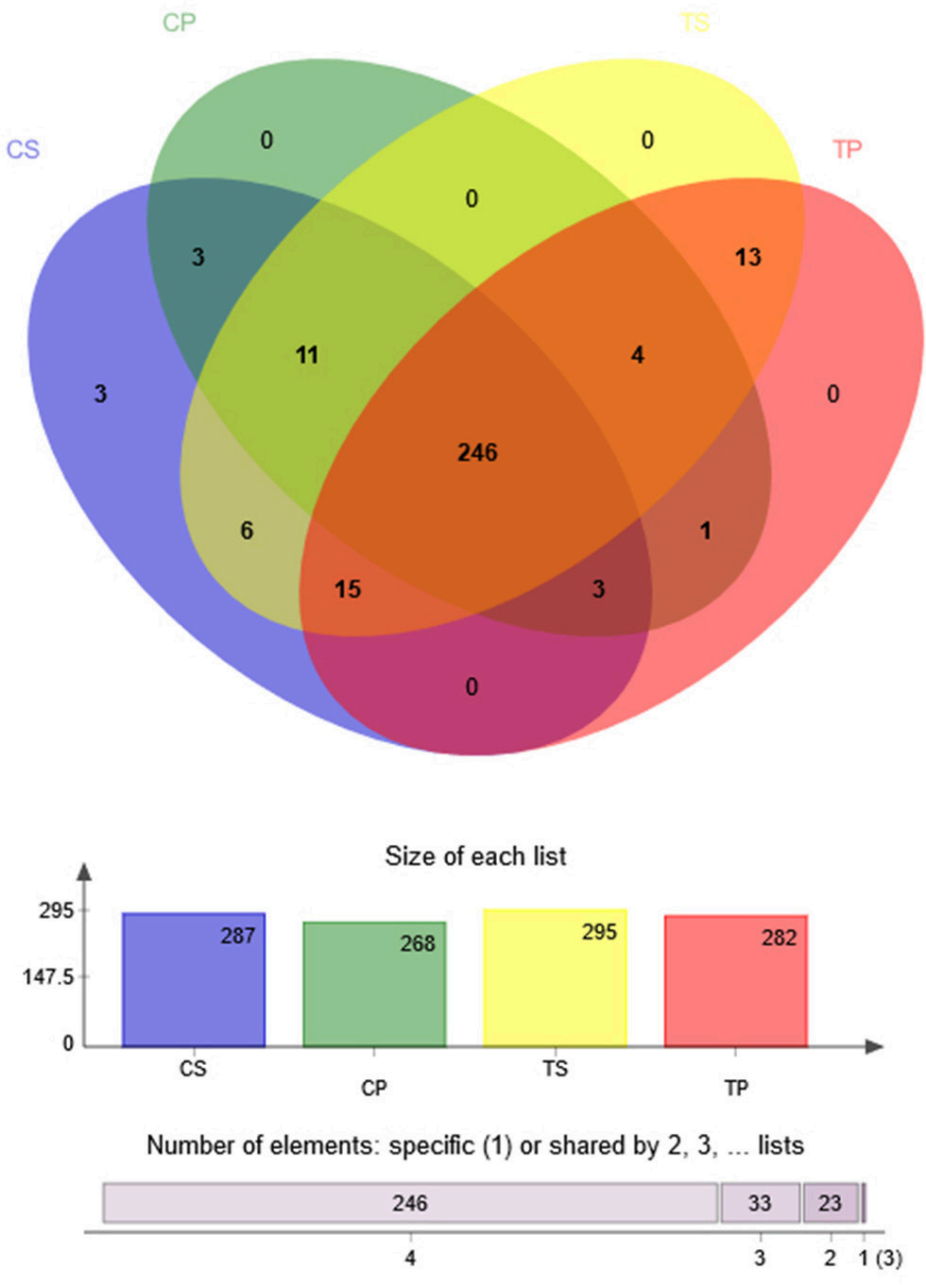
Venn diagram at the species level. Different colors represent different groups. The overlaps represent the common taxa between groups, and the non-overlapping portions represent unique taxa in each group.

A Venn diagram was made to define the core microbiome, which was detected in most individuals at the species level. We identified 287, 268, 295, and 282 species in the CP, TP, CS, and TS groups, respectively (Figure 4). Among them, 246 species were uniform, occupying 80.7% of all the species detected, indicating a steady composition of the microbiome in the dental plaque and saliva of caries patients and healthy controls. The other 59 species were not shared in all groups and were considered to be variable microbiomes. Among these variable microbiomes, 9 species could be detected only in saliva, while 1 species could be detected only in dental plaque. Similarly, 6 species could be found only in caries-free groups, while 13 species could be found only in caries-active groups (Appendix 2). Furthermore, s__Mycoplasmataceae_genomosp._P1_oral_clone_MB1_G23, s__unclassified_g__Escherichia-Shigella, and s__unclassified_g__Weissella were unique to the CS group.

### Similarity and Dissimilarity of Bacterial Compositions

**Figure 5:**
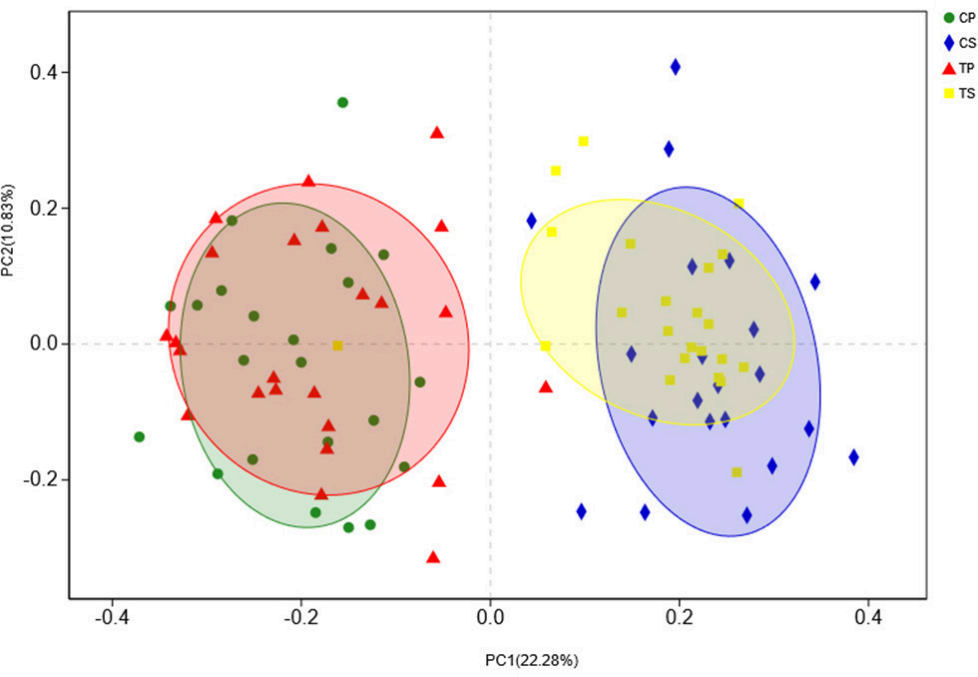
PCoA based on Bray-Curtis distances at the OTU level at 97% identity. Each sample is represented by a dot. Circles in different colors represent different groups. PC1 explained 22.28% of the variation observed, and PC2 explained 10.83% of the variation.

The similarity of the bacterial community structures among the four groups was evaluated by PCoA (Figure 5). Clear segregation was observed between samples of dental plaque and saliva, but caries-active groups showed no obvious difference from caries-free groups. This finding indicated that caries patients and healthy controls have similar oral bacterial community structures, but a distinction exists between the two niches. ANOSIM (Appendix Figure 2) and hierarchical clustering analysis (Appendix Figure 3) also demonstrated the significant distinction between different niches but no significant difference between caries-active and caries-free samples.

**Figure 6:**
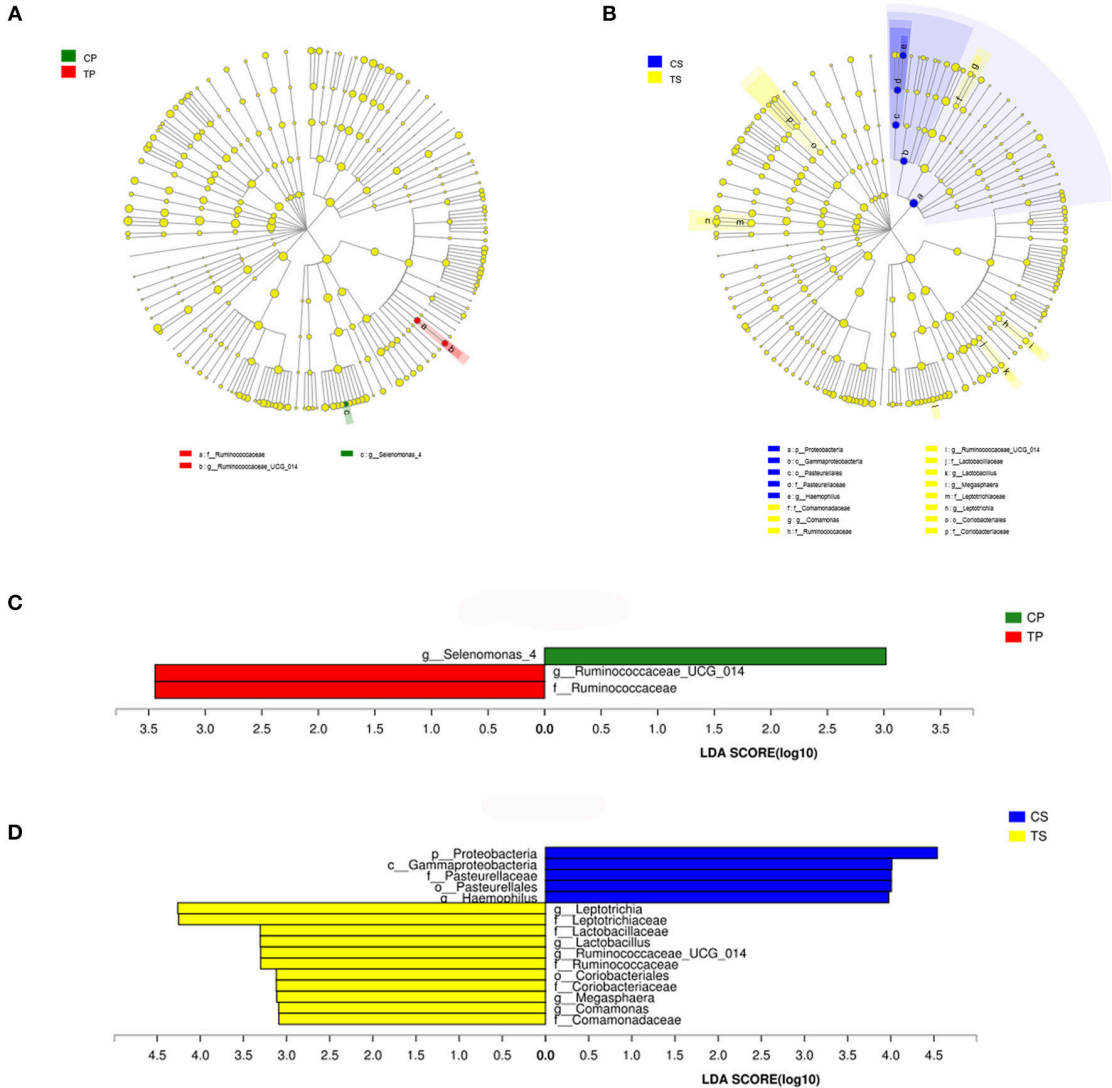
The potential biomarkers were defined by LEfSe. **(A,B)** Cladogram for taxonomic representation of significant differences between caries-active and caries-free groups. The colored nodes from the inner to the outer circles represent taxa from the phylum to genus level. The significantly different taxa are signified by different colors representing the four groups. **(C,D)** Histogram of the LDA scores for differentially abundant features among groups. The threshold on the logarithmic LDA score for discriminative features was set to 3.0.

The taxa that most likely explain the differences between caries-active and caries-free samples from different niches were defined by LEfSe. Figure 6 shows cladograms representing the potential biomarkers of different groups. At the genus level, *Selenomonas_4* was significantly enriched in the CP group, while *Ruminococcaceae_UCG_014* exhibited relatively higher abundance in the TP group. For samples collected from saliva, significant bacterial differences were also detected, with *Proteobacteria* remarkably enriched in the CS group at the phylum level. At the genus level, *Haemophilus* was significantly more abundant in the CS group, while *Comamonas, Ruminococcaceae_UCG_014, Lactobacillus, Megasphaera*, and *Leptotrichia* were most abundant in the TS group (LDA > 3, *P* < 0.05).

### Network Analysis and Function Prediction

**Figure 7:**
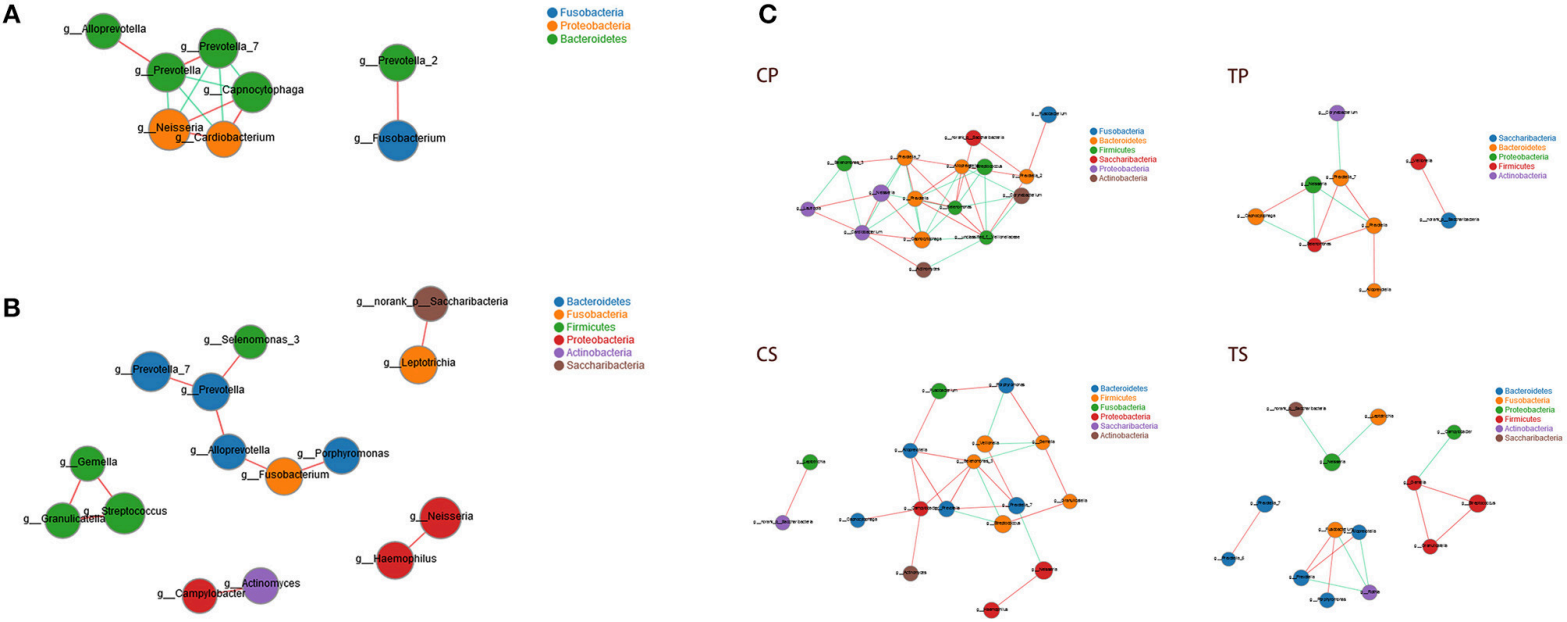
Network analysis showed interactions of 20 richest genera (|SpearmanCoef| > 0.8 and *P* < 0.01). **(A)** Bacterial interactions in dental plaque. **(B)** Bacterial interactions in saliva. **(C)** Bacterial interactions of four different groups. The size of the node is proportional to the genera abundance. Node color corresponds to phylum taxonomic classification. Edge color represents positive (red) and negative (green) correlations.

Co-occurrence analysis was performed to recognize interactions among genera in different niches. A total of 118 genera were found to have complex interactions in dental plaque, while 122 genera were found in saliva. The interactions of predominant genera are shown in Figure 7. For dental plaque microbiota, 8 genera displayed a high degree of association. Among them, *Alloprevotella, Prevotella, Prevotella_7, Capnocytophaga, Neisseria*, and *Cardiobacterium* exhibited complicated interactions with each other. For the saliva microbiota, 15 genera exhibited positive correlations. Furthermore, different groups clearly showed different bacterial correlations. Compared to caries-active groups, the microbiota of caries-free groups exhibited more complex and aggregated relationships among each other.

**Figure 8:**
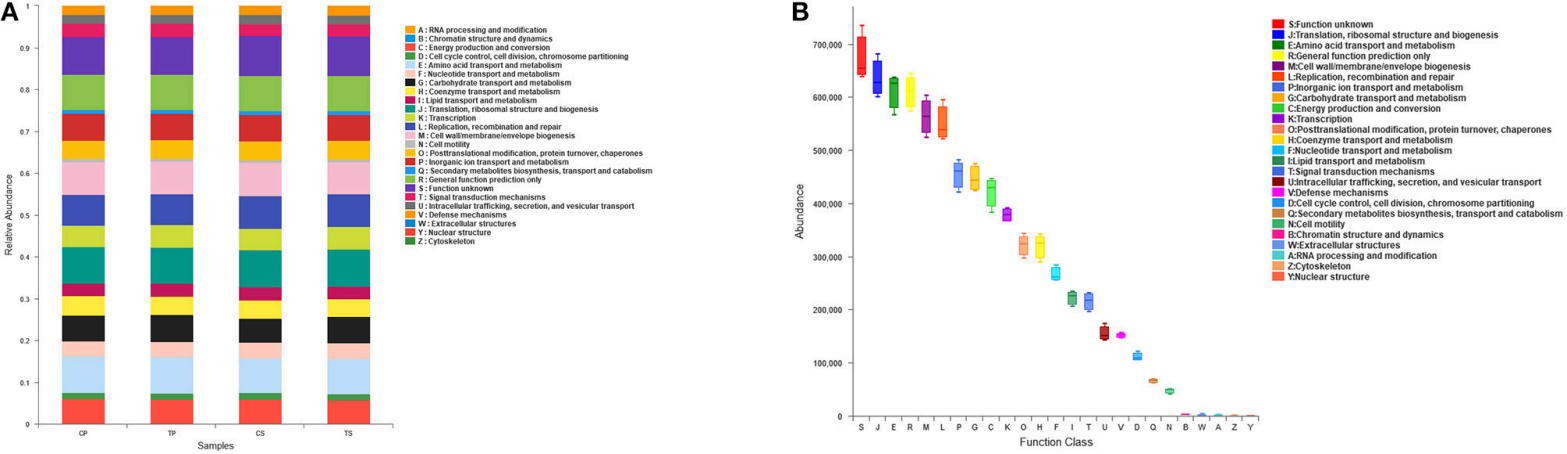
Function prediction by PICRUSt. **(A)** The compositions of COG function in the four groups. **(B)** The abundance of COG function in all samples.

The PICRUSt program was performed to help gain knowledge of the function of the microbiota present in oral specimens (Figure 8). Samples from the four groups showed similar COG profiles, indicating similar microbial functional features. The most abundant functions in all samples were: translation, ribosomal structure and biogenesis (8.77%); amino acid transport and metabolism (8.50%); general function prediction (8.45%); cell wall/membrane/envelope biogenesis (7.80%); replication, recombination and repair (7.59%); inorganic ion transport and metabolism (6.31%); carbohydrate transport and metabolism (6.18%); energy production and conversion (5.84%); and transcription (5.24%).

## Discussion

Dental caries has been a significant oral health issue in humans with a polymicrobial etiology. With the aging of the population, the elderly have increasingly suffered from dental caries in recent years. Bacteria in dental biofilms are regarded as essential in the initiation and progression of dental caries (Marsh, 1994; Chen and Jiang, 2014). Therefore, a comprehensive study of the oral microbiome is required to better understand the etiology. High-throughput techniques have provided an efficient approach to study the composition and structure of the bacterial communities associated with health and disease.

Previous studies have helped us increase our knowledge of microbial compositions in different ages. *Firmicutes* was reported to be the predominant phylum of dental plaque in young adults, while primary dentition and adult groups are most easily attacked by *Proteobacteria* (Xu et al., 2014). Some researchers found that *Firmicutes* decreased and *Proteobacteria* augmented for children, while the conclusion is totally opposite for youth and adults (Chen et al., 2017). Therefore, the oral microbial community changes along with aging, and researchers have proposed that the oral microbiome should be better investigated based on age. The current study focused on the elderly population, aiming to gain a broader view of geriatric cariogenesis.

It is generally accepted that dental caries results from multiple causes. It initiates from bacterial changes in supragingival dental plaque, which is a complicated biofilm containing a group of bacteria gathered on the tooth surfaces in a well-organized manner (Marsh, 2006). According to previous studies, it is widely acknowledged that microorganisms attached to the teeth and mucosa surfaces continue to stream into saliva, making it a reservoir of oral microbiota and a “fingerprint” of the whole oral microbiota (Fábián et al., 2008). In this study, we collected samples from dental plaque and saliva. The results can help us gain a thorough and comprehensive understanding of the bacterial community associated with dental caries of the aged.

In this study, alpha diversity indices demonstrated that the diversity and richness of the bacterial communities in caries-active groups were similar to that in caries-free groups. This finding conformed to results of some previous studies (Chen et al., 2015; Jiang et al., 2016). Other studies have indicated that the dental plaque of healthy controls show higher bacterial diversity than caries patients, indicating that more varied communities may correspond to healthier ecosystems (Li et al., 2005; Preza et al., 2008; Xiao et al., 2016). Therefore, the conclusion remains controversial. The sample size, sequencing methods, individual variations, and some other factors could affect the results. More research is required to gain further recognition. However, by comparing samples of dental plaque and saliva, significant differences could be found, showing higher diversity and lower richness of samples collected from dental plaque than saliva. This agreed with the long-standing postulation that microorganisms vary among different niches (Xu et al., 2014).

A total of 16 phyla, 29 classes, 49 orders, 79 families, 149 genera, and 305 species were detected from all the specimens. The predominant phyla were *Firmicutes, Bacteroidetes, Proteobacteria, Fusobacteria, Actinobacteria*, and *Saccharibacteria*, which is similar to previous studies (Jiang et al., 2013; Chen et al., 2015; Xiao et al., 2016), indicating a relatively steady bacterial community structure in the oral cavity. As shown in the community bar plot, the caries-active and caries-free groups showed similar community structure, suggesting that disease status may not markedly influence bacterial composition. At the genus level, most prevalent bacteria were largely consistent among the four groups but different relative abundances could be observed. These results supported the “Ecological Plaque Hypothesis,” which regards caries as a consequence of disruption in homeostasis of resident microbes, rather than the activity of specific microorganisms (Kidd and Fejerskov, 2013).

The Venn diagram showed that more than 80% of the species were uniform among groups, also indicating a steady composition of the microbiome in dental plaque and saliva of all the subjects. These shared species can be defined as “oral core microbiome,” which was first found by 454 pyrosequencing in multiple sites of three adults (Zaura et al., 2009). It was confirmed to exist and its general composition has also been studied by further researches (Hu et al., 2013; Ling et al., 2013). It is increasingly recognized that the core microbiome may play an essential role in the stability and function of the oral microecological environment. However, 59 species were considered to be variable microbiomes. Among them, 13 species could be found only in caries-active groups, while 6 species could be found only in caries-free groups. These species may be related to dental caries and health, respectively. Further studies are needed to explore the specific functions of these species and their relationships with health and disease.

The potential biomarkers of different groups are presented by LEfSe. Among these significantly different genera, *Selenomonas, Haemophilus, Lactobacillus*, and *Leptotrichia* were common in the human oral cavity. *Lactobacillus* has long been considered to be cariogenic bacteria, with acid-producing and acid-resistant abilities and high detection rate in deep caries, especially dentine and root surface caries. *Leptotrichia* often serve as the scaffold for cocci and promote the formation of dental plaque biofilm. This study indicated that *Ruminococcaceae, Comamonas*, and *Megasphaera* may also be associated with caries in the elderly.

Network analysis exhibited potential correlations of oral microbiota. Some genera belonging to *Bacteroidetes* showed active correlations with each other and with genera belonging to other phyla, suggesting that *Bacteroidetes* may play important roles in bacterial interactions of the oral microenvironment. Different niches showed distinctive bacterial interactions. Similarly, the analysis of the microbiota of caries patients and healthy controls clearly showed different relationships among oral microbiota. Caries-free groups exhibited more complex and aggregated relationships than caries-active groups, indicating that some microbial relationships may be disrupted and result in dysbiosis in caries process. It was consistent with the opinion many researchers held that changes in the nutrient status at the site due to increases in fermentable carbohydrates and the resultant acidic conditions disrupt the microbial interactions that control the balance of the microbial communities in health (Marsh and Zaura, 2017).

Function prediction was performed by the PICRUSt program based on the COG database. All groups showed similar microbial functional features, which may be influenced by the widespread core microbiome. By comparing the relative abundance of function classification, we found the metabolic functions enriched in samples, indicating vigorous microbial metabolism in the oral bacterial community.

Because of improvements in health care, retained teeth of the elderly have increased. The elderly generally suffered from gingival recession and exposed root surfaces, resulting in a high risk of root caries. Earlier studies revealed that dental plaque from root caries has different microbial compositions with that from coronal caries (Marsh and Martin, 2009). The current study aimed to investigate microbial shifts of dental caries in the elderly, but coronal caries and root caries were not separated. Further studies are needed to obtain a better understanding of microbial communities in coronal caries and root caries, respectively.

Although it is universally acknowledged that bacteria play a key role in dental caries, they are not the only factor. Microbiomes of other types can also influence disease progression. Fungal–bacterial ecological interactions have attracted researchers' attention in recent years. *Candida albicans* was reported to have a strong effect on early children's caries and root caries (Brailsford et al., 2001; Shen et al., 2002; Morales and Hogan, 2010; Klinke et al., 2014; Xiao et al., 2018). Further studies are needed to investigate other microbiomes and their relationships in the caries process. Furthermore, environmental conditions such as temperature, pH conditions, salinity, redox potential, and access to oxygen or nutrients, all have some impact on the microbial composition of biofilms (Takahashi and Nyvad, 2011). Temporary changes in the oral microbiome may result from diet, salivary flow, and long-term use of antibiotics (Sheiham, 2001; Nasidze et al., 2009).

This study focused on the polymicrobial etiology of dental caries in the elderly considering the aging of the population and the high prevalence of dental caries. It provided a thorough analysis based on the bacterial diversity and community structure of oral samples collected from the elderly with caries and health. This study expected to help find novel methods in prevention and treatment strategies. Compared to previous research on the microbiology of the elderly, this study comprehensively analyzes the microbiome of different niches and reveals the interaction and function of predominant bacteria in addition. Further studies are needed to investigate other microbiomes and their relationships in caries process, and to take environmental effect factors into consideration.

## Author Contributions

QJ contributed to the research design, data analysis and interpretation, and drafted and critically revised the manuscript. JL contributed to the research design, data acquisition, and critically revised manuscript. LC and NG contributed to data interpretation and critically revised the manuscript. DY contributed to conception and design and critically revised the manuscript. All authors gave final approval and agreed to be accountable for all aspects of this work.

### Conflict of Interest Statement

The authors declare that the research was conducted in the absence of any commercial or financial relationships that could be construed as a potential conflict of interest.
