# Supplementary material for: The Oral Microbiome in the Elderly With Dental Caries and Health
Source: Front Cell Infect Microbiol. 2019 Jan 4;8:442. doi: 10.3389/fcimb.2018.00442 (PMC6328972; doi:10.3389/fcimb.2018.00442)
Supplement: Supplementary file 2 [file Data_Sheet_1.PDF]

Supplementary Material

Appendix 1. OTU table

Appendix 2. Variable microbiomes

Appendix Figure 1. The rarefaction curves. The x axis indicates the number of reads sampled. The y axis indicates the Shannon index of OTU level. Different colors represent different groups.

Appendix Figure 2. ANOSIM based on unweighted UniFrac distances. **(A)** Results comparing the CP and the TP groups, P=0.458. **(B)** Results comparing the CS and the TS groups, P=0.105. **(C)** Results comparing dental plaque (CP&TP) and saliva (CS&TS), P=0.001.

Appendix Figure 3. Hierarchical clustering tree on OTU level. The length of the branch shows distance between two samples, different groups present in different colors.
