# Supplementary material for: The Oral Microbiome in the Elderly With Dental Caries and Health
Source: Front Cell Infect Microbiol. 2019 Jan 4;8:442. doi: 10.3389/fcimb.2018.00442 (PMC6328972; doi:10.3389/fcimb.2018.00442)
Supplement: Supplementary file 3 [file Data_Sheet_2.PDF]

Variable microbiome

Common elements in CS CP (caries-free groups):
s__unclassified_c__Alphaproteobacteria
s__Bacteroidales_genomosp._P4_oral_clone_MB2_G17
s__unclassified_g__norank_f__Leptotrichiaceae

Common elements in TS TP (caries-active groups):
s__Desulfovibrio_sp._Dsv1
s__Streptobacillus_hongkongensis
s__unclassified_o__Bacteroidales
s__Clostridiales_bacterium_canine_oral_taxon_100
s__unclassified_g__norank_f__Anaerolineaceae
s__Peptoniphilus_sp._ChDC_B134
s__Bilophila_wadsworthia
s__Scardovia_inopinata_JCM_12537
s__Brevundimonas_diminuta
s__unclassified_g__Dialister
s__unclassified_g__Acidaminococcus
s__unclassified_g__norank_f__p-2534-18B5_gut_group
s__uncultured_bacterium_g__Streptobacillus

Common elements in CS TS (saliva groups):
s__uncultured_bacterium_g__[Eubacterium]_yurii_group
s__Pseudomonas_aeruginosa_g__Pseudomonas
s__Faucicola_mancuniensis
s__Porphyromonas_sp._oral_clone_HF001
s__Deinococcus_antarcticus
s__unclassified_p__Bacteroidetes

Common elements in CP TP (dental plaque groups):
s__Petrimonas_sp._canine_oral_taxon_434

Elements only in CS:
s__Mycoplasmataceae_genomosp._P1_oral_clone_MB1_G23
s__unclassified_g__Escherichia-Shigella
s__unclassified_g__Weissella
