# Supplementary figures and images for: The Oral Microbiome in the Elderly With Dental Caries and Health
Source: Front Cell Infect Microbiol. 2019 Jan 4;8:442. doi: 10.3389/fcimb.2018.00442 (PMC6328972; doi:10.3389/fcimb.2018.00442)

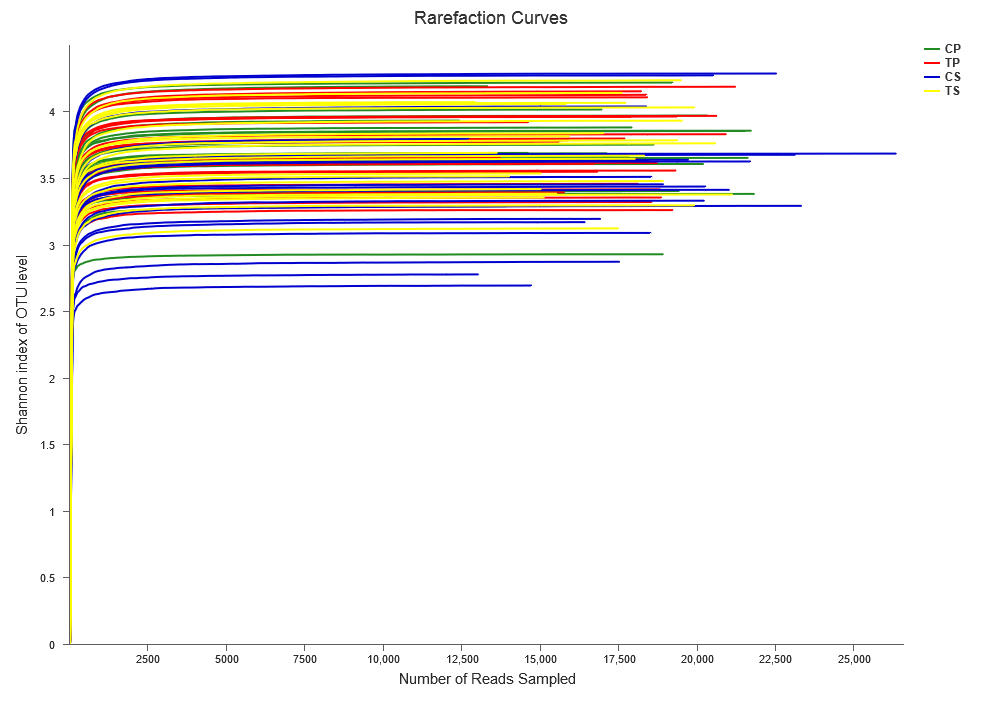

Supplement: Supplementary file 4 [file Image_1.JPEG]

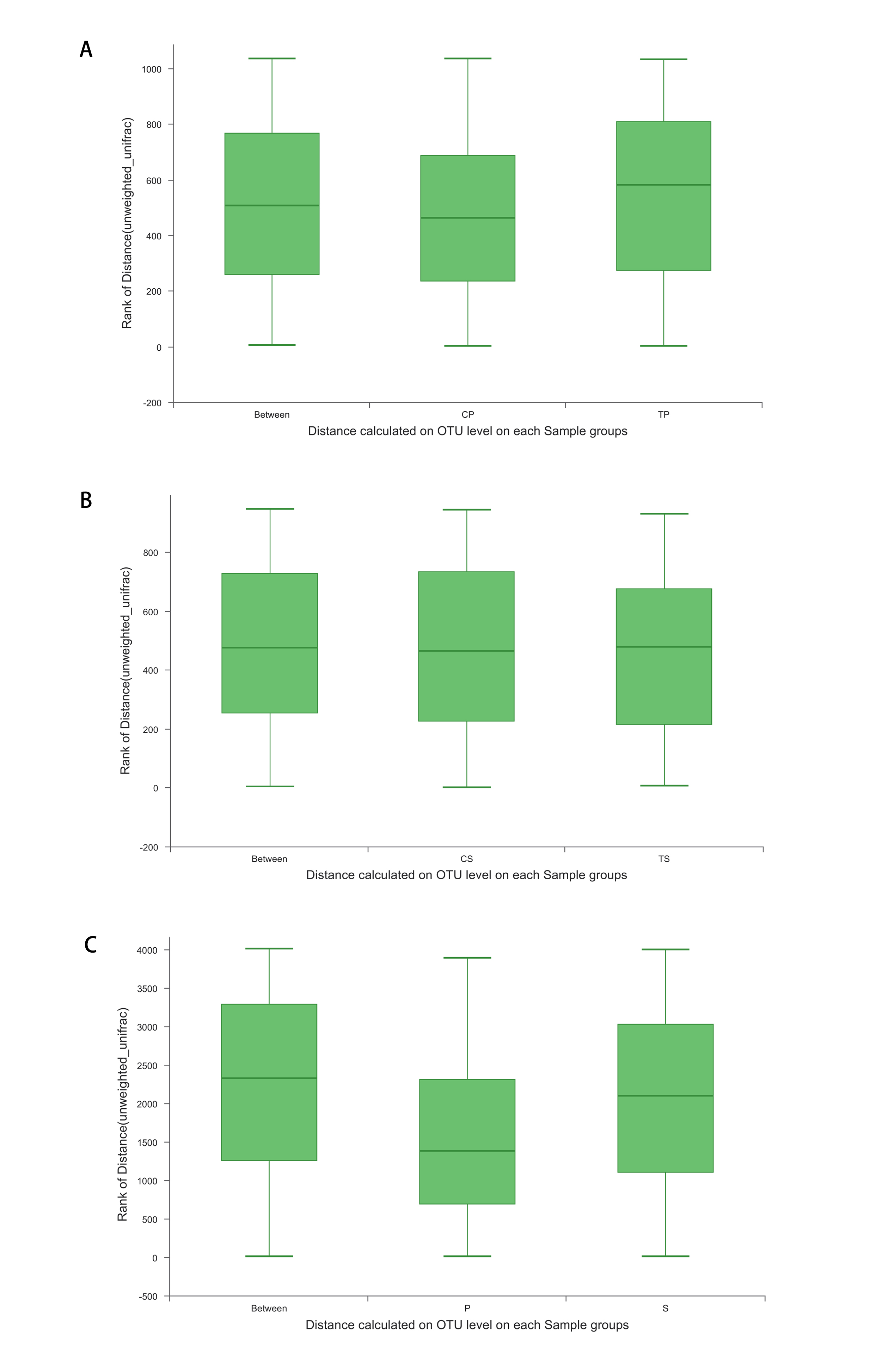

Supplement: Supplementary file 5 [file Image_2.JPEG]

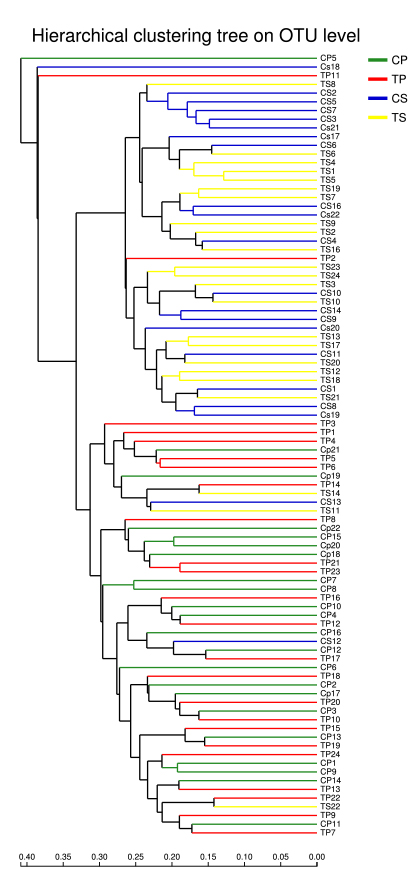

Supplement: Supplementary file 6 [file Image_3.JPEG]
